# An EMG dataset for Arabic sign language alphabet letters and numbers

**DOI:** 10.1016/j.dib.2023.109770

**Published:** 2023-11-04

**Authors:** Amina Ben Haj Amor, Oussama El Ghoul, Mohamed Jemni

**Affiliations:** Research Laboratory LaTICE, University of Tunis, Tunisia

**Keywords:** Arabic sign language, Gesture recognition, Surface Electromyography sEMG, Myo Armband

## Abstract

Nowadays, surface electromyography (sEMG) is evolving as a technology for hand gesture recognition. Detailed studies have revealed the capacity of EMG signals to access detailed information, particularly in the classification of hand gestures. Indeed, this advancement emerges as an interesting element in refining the recognition and interpretation of sign languages and exploring deeper into the phonology of signed languages. Aligned with this advancement and the need for a reliable and mobile sign language recognition system, we introduce a specialized sEMG dataset, acquired using the Myo armband. This device is adept at capturing recordings at frequencies of up to 200 Hz. The dataset focuses on the 28 letters of the Arabic alphabet and 10 digits using hand gestures, with each gesture captured into 400 frames. This considerable collection of 18,716 samples was achieved with the cooperation of three contributors, providing a varied and comprehensive range of gestural data.

Specifications Table

**Table:** 

Subject	Signal Processing
Specific subject area	Using sEMG for Sign language recognition.
Data format	Raw
Type of data	Numpy files (.npz)
Data collection	We gathered raw data utilizing the Myoware armband, equipped with eight equally spaced sensors situated around the forearm of the right hand. This setup allows capturing EMG signals at a sampling rate of 200 frames per second. During the data acquisition sessions, signs were recorded, each encapsulated within a two-second time frame. The position of the armband was kept constant to ensure accuracy and consistency in the data collected. More precisely, the main sensor was invariably situated at a predetermined, marked position on the upper part of the forearm. The data acquisition process involved 3 participants, including two men and one woman.
Data source location	Research laboratory LaTICE, University of Tunis.
Data accessibility	Repository name: AN EMG DATASET FOR ARABIC SIGN LANGUAGE ALPHABET AND NUMBERS Data identification number: 10.17632/ft9bhdgybs.2 Direct URL to data: http://doi.org/10.17632/ft9bhdgybs.2
Related research article	Amor, A. B. H., El Ghoul, O., & Jemni, M. (2022, July). Deep learning approach for sign language's handshapes recognition from EMG signals. In 2022 IEEE Information Technologies & Smart Industrial Systems (ITSIS) (pp. 1–5). IEEE.

## 1 Value of the Data

• The data was mainly recorded for sign language recognition purpose. The recorded data are particularly valuable as they offer in-depth insights into muscle activity and hand movements associated with signing. By analyzing these data, researchers can gain a deeper understanding of the physiological aspects of sign language. These detailed insights are useful in developing assistive technologies, such as real-time sign language translation devices, thereby fostering enhanced communication between deaf and hearing individuals. Beyond this, sEMG data can act as a component in human-computer interaction models, paving the way for more intuitive and user-friendly interfaces and control systems.
• The utilization of sEMG data for sign language permeates multiple domains, presenting opportunities for application and development. Foremost, the deaf and hard-of-hearing stands to accrue significant benefits, experiencing improvements in quality of life due to advancements in translation and communication devices. This data repository acts as a resource for researchers on many disciplines including computer science, linguistics, and biomechanics. It provides them the opportunity to explore the muscular movements during sign language discourse and to analyze the linguistic expressions of Arabic sign language. The multi-faceted implications of sEMG data underscore its role in advancing understanding and applications across varied sectors.
• The dataset is suitable for the training and validation of deep learning models purposed for the recognition of sign language alphabets and digits, or words signed using finger spelling, or gesture recognition algorithms. Furthermore, methodologies for data augmentation can be developed based on this data. Additionally, the availability of sEMG data motivates the development of new methodologies for data collection, processing, and analysis, promoting advancement in the field. This shared dataset can serve as benchmark or comparison groups for new research endeavors, facilitating the development and validation of innovative models.

## 2 Data Description

**Fig. 1:**
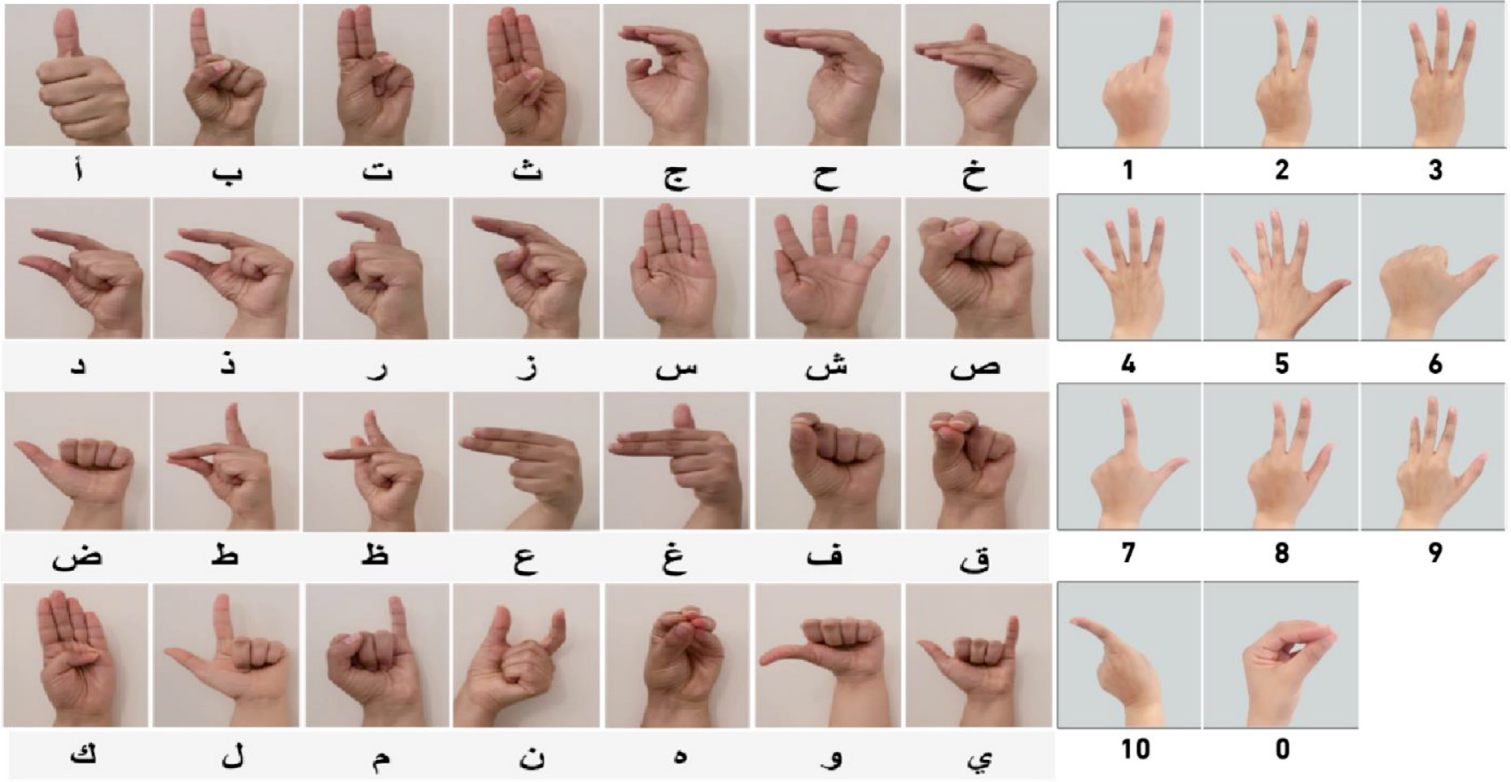
Recorded ArSL alphabet and numbers.

Sign Language Recognition (SLR) is crucial in facilitating communication for the Deaf and Hard-of-Hearing community and involves the use of modalities like Surface Electromyography (sEMG) signals and vision-based methods. The sEMG signals are paramount for capturing muscular activities and recognizing hand gestures in sign language. Despite its importance, based on our literature survey, there are limited sEMG datasets associated with sign languages. We identified four open-access datasets [1], [2], [3], [4], [5]. Addressing this, our work focuses on the creation of a comprehensive Arabic Sign Language Alphabet and digits (Fig. 1) dataset to promote research and innovation in assistive technologies, thereby enhancing accessibility and inclusivity for Arabic Deaf and Hard-of-Hearing communities.

Fig. 1, illustrates the handshapes we recorded in our dataset. We have included 28 handshapes for the Arabic alphabet and 10 more gestures for numbers from zero to ten.

**Fig. 2:**
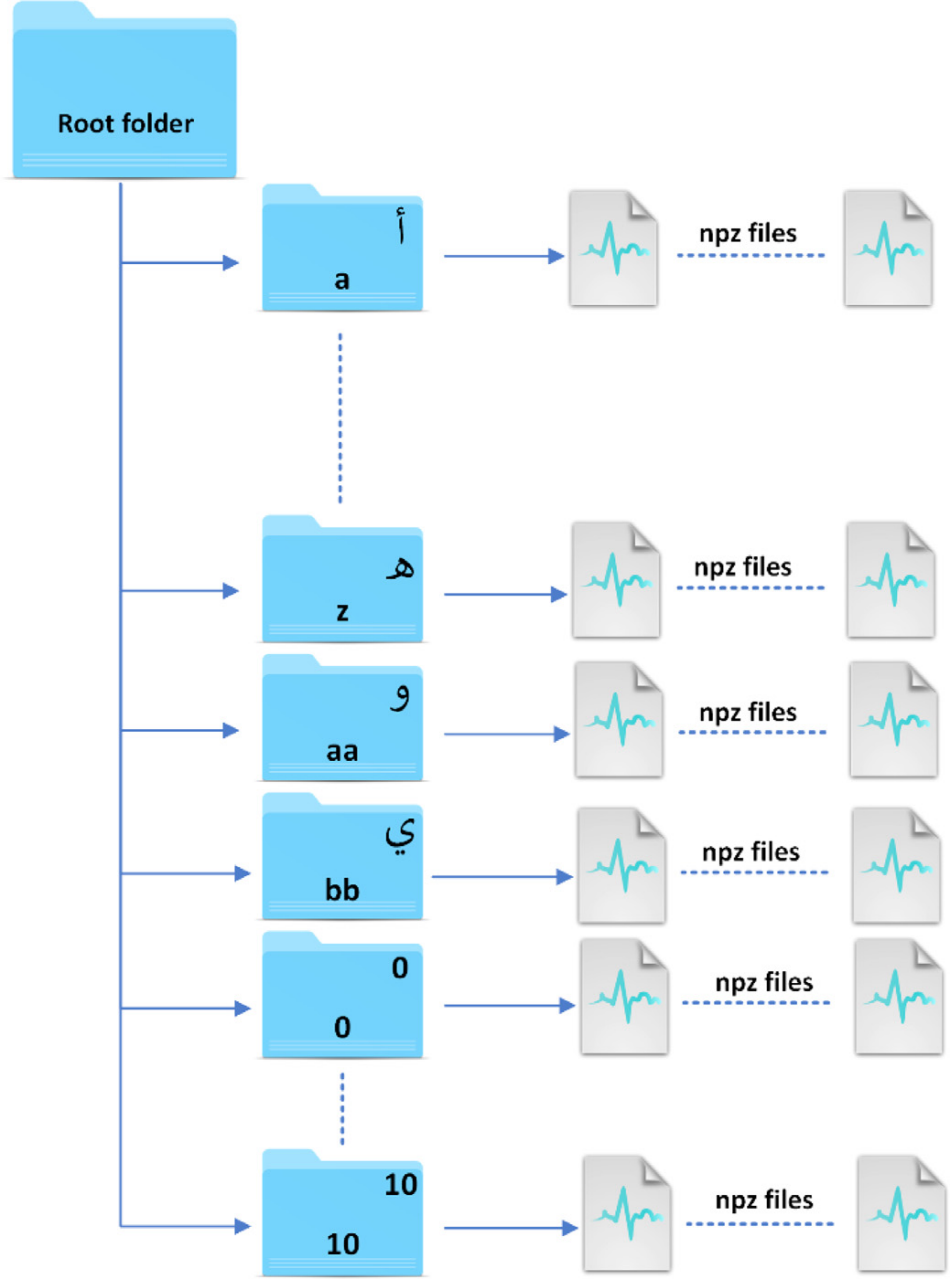
The data structure of the dataset.

Our dataset, illustrated in Fig. 2, contains 18,716 gesture files, and is organized as follows:

• The dataset is partitioned into 39 directories, each representing a distinct gesture. Accordingly, every directory consolidates all acquisitions associated with a specific gesture, with each acquisition recorded in an individual file.
• The directories employ an intuitive naming convention, reflective of the gestures they embody. For instance, gestures representing characters from “أ” to “ه” are allocated to directories labeled “a” to “z,” while distinct denominations like “aa” and “bb” are reserved for characters “و” and “ي,” respectively. Numerical gestures are logically organized within numerically labeled directories from 0 to 10, ensuring a systematic and coherent cataloging conducive to efficient navigation and extraction.
• Each directory encompasses between 350 and 650 gesture files, all saved in the .npz format.
• The .npz file format is a specialized file format employed by NumPy, a prominent library within the Python programming environment. NumPy is particularly indispensable for operations associated with arrays, matrices, and linear algebraic computations. The .npz format is meticulously designed to facilitate the storage of multiple NumPy arrays in a singular file, incorporating compression mechanisms to optimize storage efficiency.
• Each .npz file incorporates data collected from the 8 sEMG sensors of the Myo Armband, procured during an individual acquisition of the gesture, situated within a temporal window of a 2 s, and recorded at a sampling frequency of 200 Hz.

**Fig. 3:**
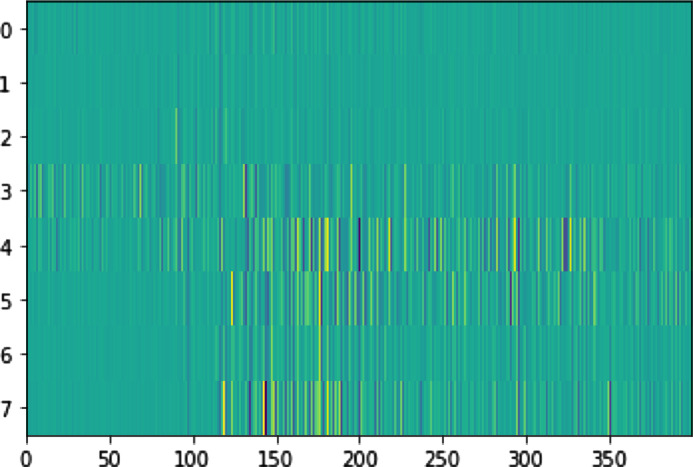
Graphical representation of a gesture data.

Fig. 3 shows the structure of a single npz file. Each npz file contains a Numpy object with the following characteristics:

• Encapsulation and Extraction: Each file encapsulates an object labeled “arr_0″. For the extraction of this object, the specified Numpy code can be employed: numpy.load(file_name)[”*arr*_0″].
• Matrix Structure and Representation: The encapsulated object, “arr_0,” embodies a matrix characterized by 8 rows and 400 columns. The succeeding figure delineates a graphical representation, illustrating a specimen of the recorded data.
• Row and Column Significance: The rows within the matrix represent the 8 sEMG sensors, whilst the columns are aligned to the 400 frames recorded during the 2 s of acquisition. Each element of this matrix represents a measurement of an sEMG signal at a specific moment during the two-second recording period, which amounts to 400×8 measurements distributed over 2 s. Consequently, this matrix provides an accurate representation of the evolution of sEMG signals over time for each channel. This organization is essential for interpreting the data in a sensor-wise and time-sequential manner.
• Data Types and Formats: The elements within the matrix are encoded as integer values, utilizing the Int64 data type.

## 3 Experimental Design, Materials and Methods

**Fig. 4:**
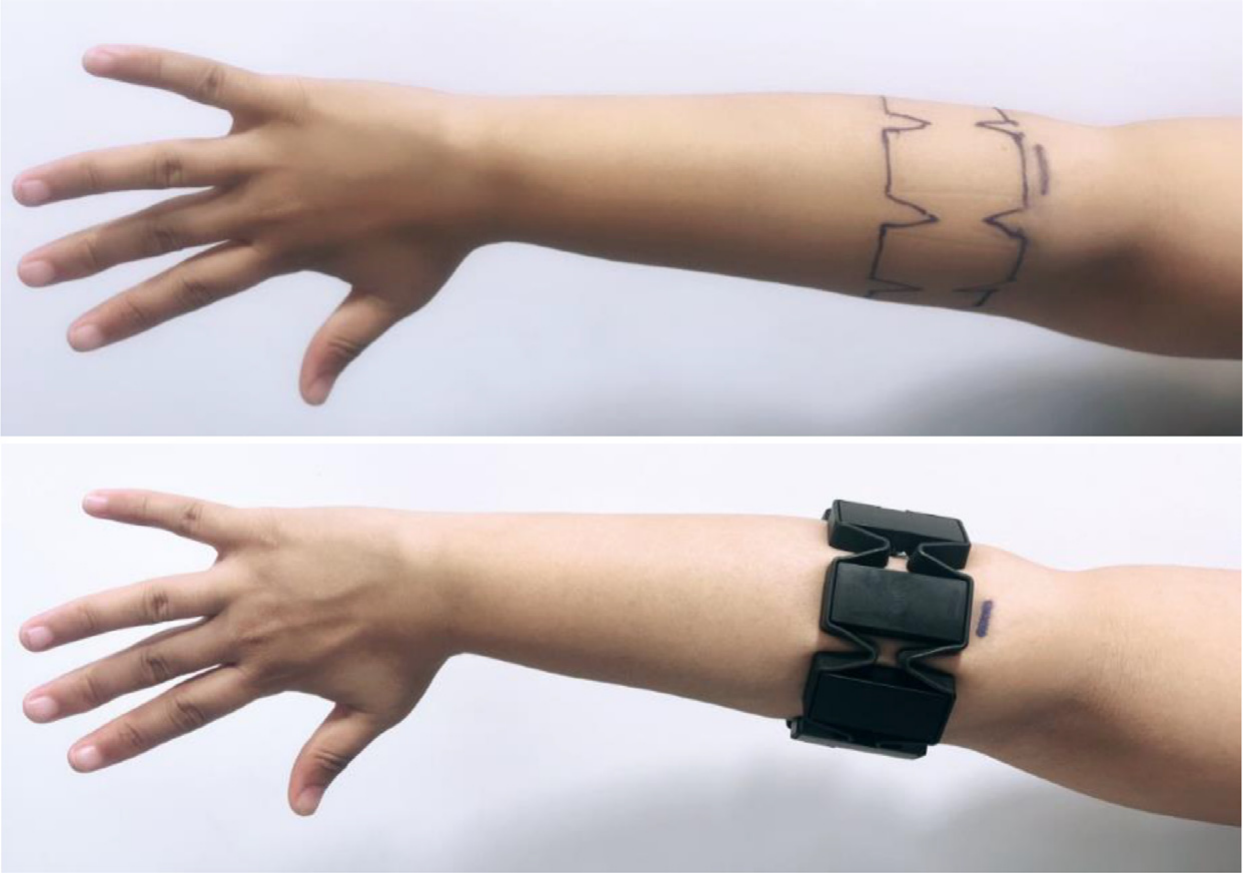
Placement of the armband.

We collected raw data from the Myo armband, which incorporates eight sensors placed at equal distances [6] around the forearm muscles in the right hand, capable of capturing real-time EMG signals at a sampling rate of 200 frames per second (Fig. 4). During the recording sessions, each gesture was performed within a two-second time window. The data were then recorded and processed as two-dimensional arrays. Subsequently, each sample resulting in a vector (8400). Fig. 3 presents an sEMG signal highlighting data related to a specific hand gesture. The Myo armband is equipped with eight sensors, each capturing unique signals that it transmits to the computer via Bluetooth. We retrieve these data by connecting the armband to a computer using the MyoConnect driver provided by Thalmic Lab. MyoSDK makes accessing the sampled data easier. For the development of our application, we chose the Visual Studio Code editor and worked with the Python language.

The data collection was carried out by three subjects consisting of two men and one woman. To ensure the rigor and authenticity of the entire process, we collaborated with experts in the field: four professionals from the deaf community at the Sports and Educational Academy for the Deaf in Tunis. Leveraging their deep expertise in visual communication and sign language, they supervised each recording session. Their role was pivotal in ensuring that each gesture was executed correctly and faithfully captured. This collaboration significantly contributed to the quality and accuracy of the collected data, thereby ensuring the relevance and reliability of our study. We embarked on an extensive data collection effort, gathering a total of 18,716 distinct samples. In order to streamline the organization, processing, and analysis of these vast datasets, each sample was meticulously recorded in an individual file.

During each data acquisition session, it was essential to maintain the armband in a constant position. Specifically, the main sensor was systematically placed on the upper part of the forearm, as depicted in Fig. 4. By ensuring a stable and uniform position of the armband throughout all sessions, we were able to minimize potential variations in EMG signals. This methodological rigor allowed us to obtain data of increased precision and reliability. Furthermore, the placement of the main sensor on the upper part of the forearm is considered a standard practice in EMG data collection for hand movements. This approach optimizes the capture of nuances in hand movements while ensuring a high level of reproducibility for each recording session.

**Fig. 5:**
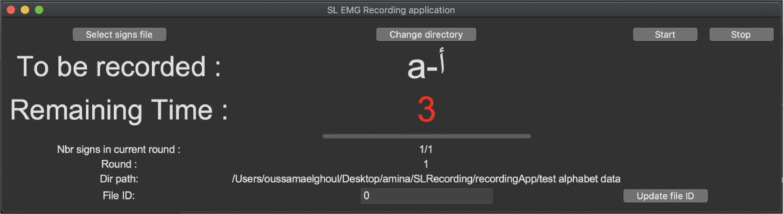
Recording application interface.

To streamline the data acquisition process, we developed an application (Fig. 5) that automates data collection, with the aim of enhancing the efficiency of recording sessions and speeding up the process. The application, as shown in Fig. 2, sequentially presents the gestures to be recorded. It provides the participant with a four-second interval to prepare for the next recording followed by 2 second to perform the gesture. During the session, the gestures start from a neutral hand position identified as 'OPEN HAND.' To assist the participant in getting ready for recording, a four-second countdown timer is displayed. Once this time has elapsed, another two-second countdown timer is activated, guiding the participant in performing the gesture.

## Limitations

EMG signals are complex and prone to numerous interferences. Indeed, various factors, such as movement artifacts, temperature fluctuations, electromagnetic radiation, and the interaction among different tissues, can disrupt these signals [7]. Therefore, preprocessing is essential to eliminate these unwanted noises.

## Ethics Statement

The authors received informed consent from the participant whose gestures were recorded in the dataset. The data, as it is made available in the public repository, maintains the anonymity of the participants, comprising only the values derived from Electromyography sensors and is devoid of any personal data.

## CRediT authorship contribution statement

**Amina Ben Haj Amor:** Conceptualization, Methodology, Software, Data curation, Writing – original draft. **Oussama El Ghoul:** Writing – original draft, Methodology, Validation. **Mohamed Jemni:** Writing – review & editing, Supervision.

## Data Availability

AN EMG DATASET FOR ARABIC SIGN LANGUAGE ALPHABET AND NUMBERS (Original data) (Mendeley Data)
